# LncRNA CAIF suppresses LPS‐induced inflammation and apoptosis of cardiomyocytes through regulating miR‐16 demethylation

**DOI:** 10.1002/iid3.498

**Published:** 2021-09-21

**Authors:** Yan Wang, Yi Zhang

**Affiliations:** ^1^ Department of Intensive Care Unit Shaanxi Provincial People's Hospital Xi'an China

**Keywords:** apoptosis, CAIF, cardiomyocyte, methylation, miR‐16, sepsis

## Abstract

**Background:**

The long noncoding RNA, cardiac autophagy inhibitory factor (CAIF), and microRNA (miR)‐16 are reported to be involved in lipopolysaccharide (LPS)‐induced inflammatory responses and cell apoptosis in many diseases. Herein, we investigated the interaction between CAIF and miR‐16 in sepsis‐induced chronic heart failure (CHF).

**Methods:**

The expression of CAIF and miR‐16 in plasma samples from sepsis‐induced CHF patients (*n* = 60) and healthy controls (*n* = 60) were measured using quantitative reverse‐transcription polymerase chain reaction (qRT‐PCR). The correlations between CAIF and miR‐16 across plasma samples from patients with sepsis‐induced CHF and healthy controls were analyzed using linear regression. The messenger RNA (mRNA) levels of inducible nitric oxide synthase, C‐C motif chemokine 2 (CCL2), growth‐regulated alpha protein (CXCL1), and interleukin‐6 (IL‐6) were evaluated using qRT‐PCR while nuclear factor κB activation was evaluated using luciferase assay.

**Results:**

The expression levels of CAIF and miR‐16 were downregulated in the plasma of sepsis‐induced CHF patients and were positively correlated in these patients. In cardiomyocytes, LPS treatment dose‐dependently decreased CAIF and miR‐16 levels. CAIF overexpression increased miR‐16 expression by demethylating miR‐16. CAIF and/or miR‐16 overexpression suppressed LPS‐induced CCL2, CXCL1, and IL‐6 expression at both the mRNA and protein levels. Analysis of cell apoptosis and western blot analysis showed that CAIF and/or miR‐16 overexpression inhibited LPS‐induced cardiomyocyte apoptosis by reducing Bax and cleaved caspase 3 levels and enhancing Bcl‐2 levels.

**Conclusion:**

Our study is the first to report the abnormal expression of CAIF and miR‐16 in heart disease. CAIF plays a protective role in sepsis‐induced CHF by inhibiting cardiomyocyte apoptosis and inflammation, possibly by regulating miR‐16 demethylation.

## Graphical abstract

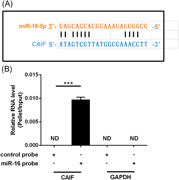

**Abbreviations**
CAIF: cardiac autophagy inhibitory factor
CCL2: C‐C motif chemokine 2
CHF: chronic heart failure
CXCL1: growth‐regulated alpha protein
IL‐6: interleukin‐6
iNOS: inducible nitric oxide synthase
LPS: lipopolysaccharides
LncRNAs: long noncoding RNAs
NF‐κB: nuclear factor κB
qRT‐PCR: quantitative reverse‐transcription polymerase chain reaction

## 1 INTRODUCTION

Upon infection, the human body releases chemicals into the blood to fight against invaders. However, in some cases, the body's responses to these chemicals may be out of control, leading to sepsis.^1^, ^2^ Sepsis causes damage to different organs.^3^ Without timely and proper treatment, organ damage can become irreversible, leading to a high mortality rate.^4^ It has been estimated that more than 40% of patients with severe sepsis die of this disease.^5^ In some cases, chronic heart failure (CHF) can be induced by severe sepsis.^6^, ^7^ At present, the molecular mechanisms underlying sepsis‐induced CHF remains unclear, making the development of new therapeutic methods a challenge.

The development of sepsis and sepsis‐induced organ failure has been reported to involve multiple molecular pathways.^8^ Increased understanding of the functionality of these molecular pathways provides novel insights into the development of targeted therapy, which aims to improve the conditions of sepsis by regulating sepsis‐related gene expression.^9^, ^10^ Lipopolysaccharides (LPS)‐induced inflammatory responses play a detrimental role in sepsis.^11^ It has been well established that different types of noncoding RNAs (ncRNAs), such as microRNAs (miRNAs) and long ncRNAs (lncRNAs, >200 nt), are involved in LPS‐mediated inflammation,^12^ indicating their potential participation in sepsis and sepsis‐induced CHF.

LncRNA cardiac autophagy inhibitory factor (CAIF) is a newly discovered lncRNA that reduces myocardial infarction by inhibiting p53‐mediated myocardial transcription,^13^ suggesting that CAIF may play a protective role in heart diseases. CAIF could also inhibit the LPS‐induced inflammatory responses by regulating miR‐1246 expression in osteoarthritis,^14^ indicating that CAIF may be an important resistor of inflammation. Therefore, the role of CAIF in inflammation‐related diseases, including CHF, needs to be investigated.

Previous studies have demonstrated that miR‐16 plays a crucial role in anti‐inflammation in some diseases, such as atherosclerosis,^15^ multiple myeloma,^16^ and osteoarthritis.^17^ Moreover, several studies have revealed that miR‐16 could inhibit LPS‐induced inflammation by targeting DOCK2,^18^ PI3K,^19^ and CXCR3.^20^ Importantly, miR‐16 is regarded as a potential nuclear factor κB (NF‐κB)‐related miRNA that resists LPS‐induced inflammation by mediating CD40 in myocarditis.^21^ However, whether there is a regulatory relationship between miR‐16 and CAIF and whether these two RNAs play roles in CHF require further investigation. In this study, the findings of the bioinformatics analysis revealed that miR‐16 could bind to CAIF. Therefore, we further explored their expression in CHF patients and cardiomyocyte cells, and their interactions and regulated pathways in cardiomyocyte cells, with the hope of providing novel molecular diagnostic biomarkers and targets for CHF treatment.

## 2 MATERIALS AND METHODS

### 2.1 Study patients and plasma samples

A total of 60 sepsis‐induced CHF patients (35 males and 25 females; ages 42–67 years, 53.6 ± 6.7 years) who were consecutively transferred from the Respiratory Department to the Intensive Care Unit of Shaanxi Provincial People's Hospital between January 2017 and December 2019 were enrolled in the study if they: (1) over 18 years old, (2) newly diagnosed with sepsis‐induced CHF. Patients were excluded if (1) they were nonheart failure patients, (2) had other severe clinical disorders, such as cancers, diabetes, and other severe infections; (3) had CHF induced by other pathological factors; and (4) had been treated for any clinical disorders within 3 months before the study. In addition, 60 gender‐ and age‐matched healthy controls (gender: 35 males and 25 females; age: 42–67 years, 53.6 ± 6.8 years) who underwent systemic physiological examination at the Health Center of Shaanxi Provincial People's Hospital and had normal physiological functions were included. This study was approved by the Ethics Committee of our hospital and was designed as a randomized controlled trial (RCT) and non‐RCTs. Written informed consent was obtained from all patients. Blood (5 ml) samples were extracted from the elbow veins of all participants before therapy after fasting overnight and at the end of disease progression. Blood was mixed with ethylenediaminetetraacetic acid, followed by centrifugation for 10 min at 1200*g* to separate the plasma. Fresh plasma samples were stored in a liquid nitrogen tank.

### 2.2 Cell lines and transient transfection

The human cardiomyocyte cell line, AC16 (Sigma‐Aldrich), was used as a model that maintains cardiomyocyte characteristics and has been widely used to study normal development and pathological changes at the cellular and molecular levels. Cells were cultured in Dulbecco's modified Eagle's medium containing 12% fetal bovine serum and 1% penicillin and streptomycin at 37°C in a 5% CO_2_ incubator with 95% humidity, and harvested at 85% confluence to perform the subsequent assays.

The CAIF expression vector was constructed with pcDNA3.1 (Invitrogen) as the backbone. Negative control (NC) miRNA (5ʹ‐CGUUUGGCUAGUCAGUGUGGCA‐3ʹ) and miR‐16 mimic (5ʹ‐UAGCAGCACGUAAAUAUUGGCG‐3ʹ) were purchased from Sigma‐Aldrich. AC16 cells were transfected with the CAIF expression vector (10 nM) or miRNA (40 nM) using Lipofectamine 2000 (Invitrogen), following the manufacturer's instructions. Control (C) cells were left untransfected while negative control (NC) cells were transfected with NC miRNA or empty vector. Subsequent experiments were performed 48 h later. To mimic septic conditions, cells were incubated with 10 µg/ml LPS for 48 h before transfection.

### 2.3 RNA preparation

Total RNA was extracted from AC16 cells using RiboZol (Invitrogen). For LPS treatment, AC16 cells were incubated with LPS (Sigma‐Aldrich) from *Escherichia coli* O111:B4 at doses of 0, 2, 5, and 10 µg/ml for 48 h before use. All RNA samples were digested with DNase I (Invitrogen) to completely remove genomic DNA.

### 2.4 RNA pull‐down assay

For miRNA pull‐down, AC16 cells were transfected with biotinylated miR‐16 (miR‐16 probe) or a control probe (Genescript), and harvested in lysis buffer (20 mM Tris pH 7.5, 100 mM KCl, 5 mM MgCl_2_, 0.5% NP‐40, and 1 U/µl recombinant RNase inhibitor) (TaKaRa). After pretreatment with DNase I and heating at 65°C for 5 min followed by submersion in an instant ice bath, RNAs were incubated with streptavidin‐coated magnetic beads (New England BioLabs, S1420S) at 4°C for 4 h. After incubation, the beads were washed twice with lysis buffer, and RNA was extracted with TRIzol (Invitrogen) and used to detect lncRNA CAIF expression by quantitative reverse‐transcription polymerase chain reaction (qRT‐PCR).

### 2.5 qRT‐PCR assay

The QuantiNova Reverse Transcription Kit (Qiagen) was used to reverse transcribe total RNA samples into complementary DNA (cDNA) samples. Using cDNA samples as the template, the QuantiFast SYBR Green PCR Kit (Qiagen) was employed for qPCR to measure CAIF levels, with 18S ribosomal RNA (rRNA) used as the internal control.

To measure mature miR‐16 expression levels, the addition of poly (A), miRNA reverse‐transcription, and qRT‐PCR were performed using the All‐in‐One miRNA qRT‐PCR reagent kit (GeneCopoeia). To measure the levels of inducible nitric oxide synthase (iNOS), C‐C motif chemokine 2 (CCL2), growth‐regulated alpha protein (CXCL1), and interleukin‐6 (IL‐6) mRNAs, AC16 cells were treated with 5 μg/ml LPS for 24 h. RNA extraction, reverse‐transcriptase (RT) reaction, and quantitative real‐time PCR were performed as described above. The PCR thermal cycling conditions were as follows: (i) an initial step at 94°C for 5 min, (ii) 30 cycles of 94°C for 1 min, 60°C for 1 min, and 74°C for 1 min 30 s; and (iii) a final 7 min at 74°C. The reaction was performed in a 15 μl system containing 100 ng cDNA, 0.39 U Taq DNA polymerase (Promega), 2 mM MgCl_2_, and 400 nM primer pairs. The primers used for qPCR included CAIF forward 5ʹ‐CTTCACTCCTGCAAATGTGT‐3ʹ and reverse 5ʹ‐TTATAGTGGGATGGGCAGTT‐3ʹ, 18S rRNA forward 5ʹ‐CTACCACATCCAAGGAAGC‐3ʹ and reverse 5ʹ‐TTTTCGTCACTACCTCCCCG‐3ʹ. iNOS forward 5ʹ‐TCTGCGCCTTTGCTCATGAC‐3ʹ and reverse 5‐TAAAGGCTCCGGGCTCTG‐3; CCL2 forward 5‐TGAGGTGGTTGTGGAAAAGG‐3′ and reverse 5‐CCTGCTGTTCACAGTTGCC‐3; CXCL1 forward 5‐TGGGGACACCTTTTAGCATC‐3 and reverse 5‐GCCCATCGCCAATGAGCTG‐3; IL‐6 forward 5‐CCAGAGATACAAAGAAATGATGG‐3′ and reverse 5‐ACTCCAGAAGACCAGAGGAAAT‐3; GAPDH forward 5‐ACTCCACTCACGGCAAATTC‐3 and reverse 5‐CCTTCCACAATGCCAAAGTT‐3, miR‐16 forward 5ʹ‐UAGCAGCACGUAAAUAUUG‐3ʹ and miR‐16 reverse from the kit, as well as U6 forward and reverse primers from the kit. Three replicates were performed for each experiment, and the 2^‐∆∆Ct^ method was used to analyze the data.

### 2.6 Methylation‐specific PCR (MSP)

Genomic DNA was extracted from AC16 cells using the Monarch Genomic DNA Purification Kit (NEB). After bisulfite conversion using the DNA Methylation‐GoldTM kit (ZYMO Research), samples were used as the template for MSP using 2xTaq Taq master mix (NEB) to analyze miR‐16 gene methylation. M‐MSP forward GGGGCGCGTATCGCGG reversal GCCAATATTTACGTGCTGCTA 55°C/35x/2 mM.

U‐MSP forward GGGGTGTGTATTGTGG reversal CTCCGCCAATATTTACGTGCTGCTA 57°C/35x/1.5 mM.

### 2.7 Luciferase assay

AC16 cells (2 × 10^5^ cells) were assigned to six groups and transfected with either (i) 0.2 μg pNF‐κB‐Luc (Promega) and 0.2 μg pRL‐TK (Promega), (ii) 0.2 μg pNF‐κB‐Luc, 0.2 μg pRL‐TK, and 0.5 μg pcDNA3, (iii) 0.2 μg pNF‐κB‐Luc, 0.2 μg pRL‐TK, and 0.5 μg pcDNA3.1‐CAIF, (iv) 0.2 μg pNF‐κB‐Luc, 0.2 μg pRL‐TK, and 2 μg NC‐miRNA, (v) 0.2 μg pNF‐κB‐Luc, 0.2 μg of pRL‐TK and 2 μg miR‐16 mimic, and (vi) 0.2 μg pNF‐κB‐Luc, 0.2 μg pRL‐TK, 0.5 μg pcDNA3.1‐CAI, and 2 μg miR‐16 mimic using the Neon Transfection System (Life Technologies). After 36 h, transfected cells were treated with or without 5 μg/ml LPS for 6 h. Luciferase assays were performed using the Dual‐Luciferase Reporter Assay System (Promega).

### 2.8 Nitric oxide (NO) measurement

AC16 cells were seeded in a 24‐well plate at 2 × 10^5^ cells per well and incubated with LPS (5 μg/ml) for 24 h. The culture medium was collected, and the level of nitrite was measured using the Griess reaction, as previously described.^15^

### 2.9 Western blot analysis

AC16 cells were transfected with pcDNA3.1, pcDNA3.1‐CAI, NC‐miRNA, miR‐16 mimic, or pcDNA3.1‐CAI plus miR‐16 mimic. At 24 h posttransfection, cells were stimulated with 5 μg/ml LPS for 24 h. Cells were then collected and counted. Cells (8 × 10^5^) were dissolved for nucleoprotein extraction using a commercial kit (Nucleoprotein Extraction Kit), and 5×10^5^ cells were dissolved for total protein extraction. The extracted proteins were quantified using a BCA kit (Sangon). After denaturation in boiled water for 10 min, proteins were separated electrophoretically on 10% sodium dodecyl sulphate–polyacrylamide gel electrophoresis gels and transferred onto polyvinylidene fluoride membranes. The membranes were blocked in tris‐buffered saline (TBS) containing 5% milk (nonfat) for 1 h at room temperature, and subjected to primary blotting using rabbit primary antibodies against NF‐κB p65 (1:500; C22B4; Cell Signaling Technology), H3 (1:1000; #9715; Cell Signaling Technology), Bax (1:1000; ab182733; Abcam), cleaved caspase 3 (1:1000; ab49822; Abcam), procaspase‐3 (1:1000; ab32150; Abcam), Bcl‐2 (1:1000; ab59348; Abcam), and GAPDH (1:8000; 60004‐1‐Ig; Proteintech), respectively, overnight at 4°C. Secondary blotting was performed using HRP‐labeled goat secondary antibody (IgG) (1:1000; ab6721; Abcam) for 1 h at room temperature. Signals were developed by incubating the membranes with Amersham ECL Western Blot Detection Reagent (GE Healthcare) and processed using ImageJ v1.48 software.

### 2.10 Enzyme‐linked immunosorbent assay (ELISA)

AC16 cells were seeded in a 24‐well plate with 2 × 10^5^ cells per well after transfection, as described in the Western blot section. Thereafter, cells were stimulated with 5 μg/ml LPS for 24 h. Culture media were collected, and CCL2 and IL‐6 contents were determined using Immunoassay Kits (eBioscience), while CXCL1 content was determined using a CXCL1 ELISA kit (R&D Systems).

### 2.11 Cell apoptosis assay

AC16 cells harvested at 48 h posttransfection were subjected to a cell apoptosis assay using the Fluorescein isothiocyanate (FITC)‐Annexin V Apoptosis Detection Kit with propidium iodide (PI) (BioLegend). Briefly, AC16 cells were incubated with 5 µg/ml LPS for 24 h and washed with cold phosphate‐buffered saline. The AC16 cells were subsequently incubated with FITC‐Annexin V and PI for 20 min in the dark. Finally, the apoptotic cells (early apoptosis) were separated by flow cytometry.

### 2.12 Statistical analysis

All experiments were performed in triplicate. Data are expressed as mean ± *SD* and analyzed using GraphPad Prism 6 (GraphPad). Comparisons between two groups and among multiple groups were performed using unpaired *t* test and analysis of variance Tukey test, respectively. Correlations were analyzed by linear regression. A value of *p* < .05 was considered statistically significant.

## 3 RESULTS

### 3.1 CAIF and miR‐16 expression levels were downregulated in the plasma of sepsis patients

**Figure 1:**
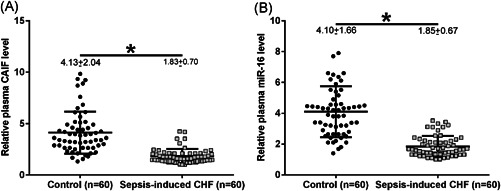
The expression levels of CAIF and miR‐16 were downregulated in the plasma of sepsis patients. CAIF (A) and miR‐16 (B) expression in plasma samples from sepsis patients (*n* = 60) and healthy controls (*n* = 60) were measured by RT‐qPCR. PCR was repeated three times, and the mean values were presented and compared. Numbers indicated above the individual scatterplots represent the mean ± *SD*. *p* values were calculated by the Mann–Whitney test. CAIF, cardiac autophagy inhibitory factor; CHF, chronic heart failure; miR, microRNA; qRT‐PCR, quantitative reverse‐transcription polymerase chain reaction. *p < .05

The expression of CAIF and miR‐16 in plasma samples in sepsis patients (*n* = 60) and healthy controls (*n* = 60) were measured by qRT‐PCR. Compared with the control group, the expression of plasma CAIF and miR‐16 were significantly decreased in sepsis patients by 55% (Figure 1A, *p* < .05) and 53% (Figure 1B, *p* < .05), respectively.

### 3.2 CAIF and miR‐16 expression levels were positively correlated across plasma samples in sepsis‐induced CHF patients

**Figure 2:**
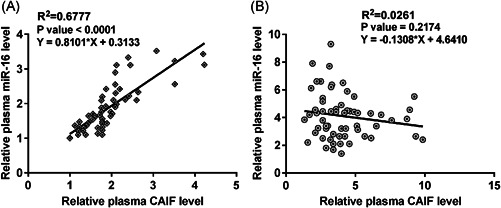
The expression of CAIF and miR‐16 were positively correlated across plasma samples from sepsis patients. The correlations between CAIF and miR‐16 expression across plasma samples from patients with sepsis (A) and healthy controls (B) were analyzed using linear regression. CAIF, cardiac autophagy inhibitory factor; miR, microRNA

The correlation between CAIF and miR‐16 expression across plasma samples from sepsis patients (*n* = 60) and healthy controls (*n* = 60) was analyzed using linear regression. The expression of plasma CAIF and miR‐16 was significantly and positively correlated across plasma samples from sepsis patients (*R* ^2^ = 0.6777, *p* < .0001, Figure 2A). However, no significant correlation was found between the expression of plasma CAIF and miR‐16 in plasma samples from healthy controls (*R* ^2^ = 0.0261; *p* > .05, Figure 2B).

### 3.3 MiR‐16 was a regulatory target of CAIF

**Figure 3:**
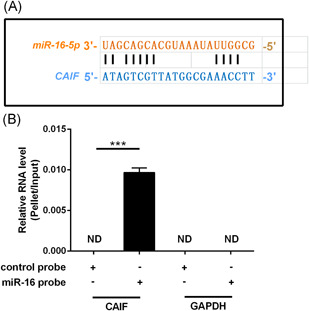
LncRNA CAIF directly interacted with miR‐16 in AC16 cells. RNA interaction was predicted using the IntaRNA (http://rna.informatik.uni-freiburg.de/IntaRNA/) database, which showed that lncRNA CAIF might bind to miR‐16 (A). RNA pulldown was conducted to confirm this interaction, showing that miR‐16 could only be amplified by qRT‐PCR after immunoprecipitation with the miR‐16 probe, but not by the control probe (B). The experiments were repeated in triplicate, and the data are presented as the mean. CAIF, cardiac autophagy inhibitory factor; GAPDH, glyceraldehyde 3‐phosphate dehydrogenase; lncRNA, long noncoding RNA; miR, microRNA; qRT‐PCR, quantitative reverse‐transcription polymerase chain reaction. *p < .05

By investigating the RNA interactions detected by IntaRNA (http://rna.informatik.uni-freiburg.de/IntaRNA/), we found that CAIF might bind to miR‐16 (Figure 3A). To confirm the interaction between miR‐16 and CAIF, we performed biotin‐avidin pull‐down assays. After transfection with miR‐16 probes into AC16 cells for 48 h, we used streptavidin‐coated magnetic beads to pull‐down biotinylated miR‐16 and measured the co‐precipitated CAIF by qRT‐PCR. CAIF could only be detected in the precipitate pulled down by the miR‐16 probe and was undetectable in the product precipitated by the control probe (Figure 3B), indicating that miR‐16 could directly interact with CAIF in AC16 cells.

### 3.4 CAIF overexpression decreased miR‐16 expression by demethylation

**Figure 4:**
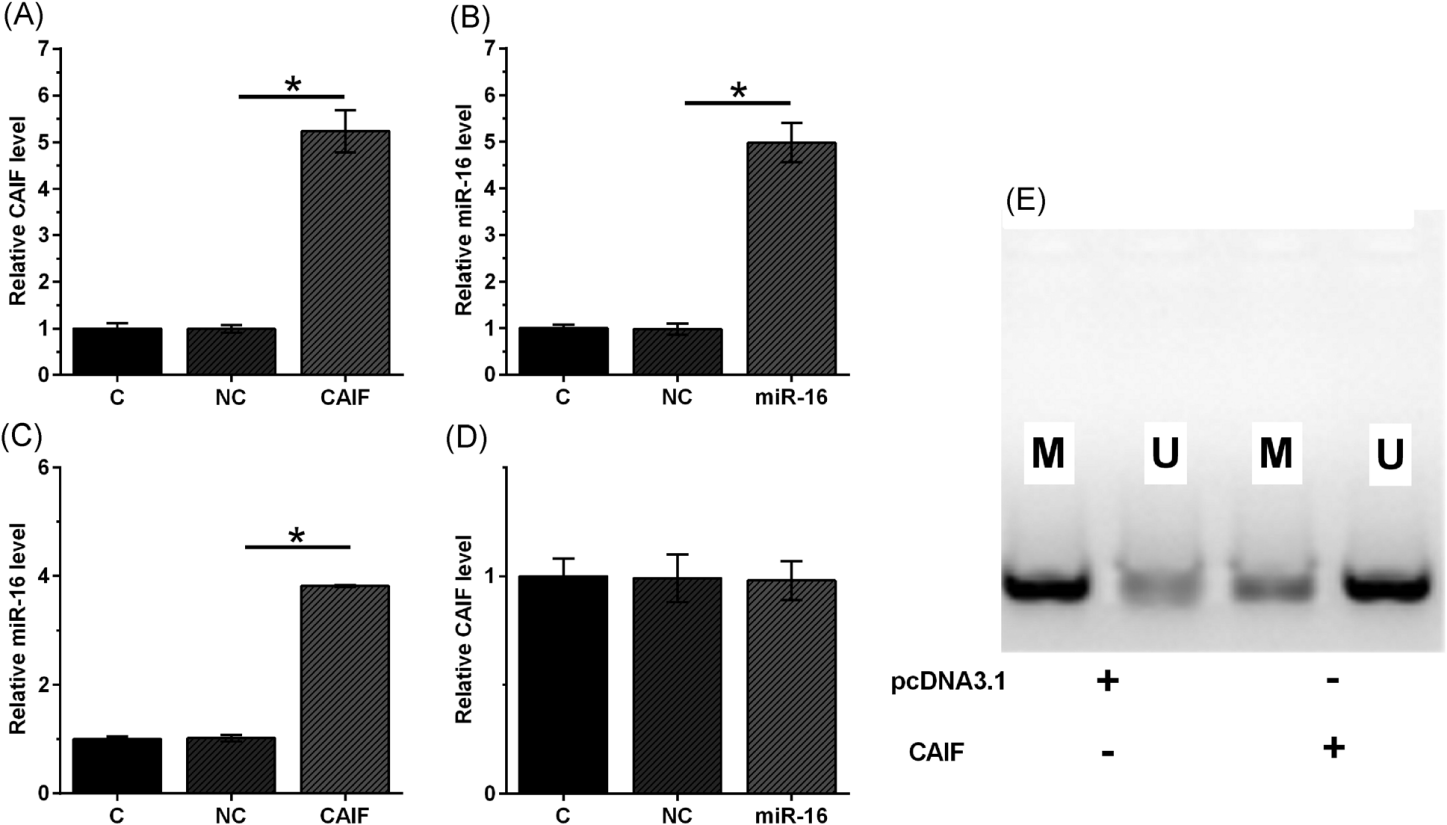
CAIF overexpression increased miR‐16 expression by increasing demethylation. AC16 cells were transfected with the CAIF expression vector or miR‐16 mimic, and overexpression was confirmed by RT‐qPCR at 48 h posttransfection (A). The effects of CAIF overexpression on miR‐16 (B) and the effects of miR‐16 overexpression on CAIF (C) were analyzed by qRT‐PCR. Methylation‐specific PCR was performed to analyze the effects of CAIF overexpression on miR‐16 gene methylation (D). The experiments were repeated in triplicate, and the mean ± *SD* values were compared. C, control cells without transfection; CAIF, cardiac autophagy inhibitory factor; M, methylated PCR product; miR, microRNA; NC, cells transfected with empty vector or NC miRNA; qRT‐PCR, quantitative reverse‐transcription polymerase chain reaction; U, unmethylated PCR product. *p < .05

AC16 cells were transfected with the CAIF expression vector or miR‐16 mimic, and the overexpression of CAIF and miR‐16 (>fourfold) in AC16 cells was confirmed by qRT‐PCR at 48 h posttransfection (Figure 4A,B, *p* < .05). Compared with the C and NC groups, CAIF overexpression increased miR‐16 expression (3.8‐fold, Figure 4C, *p* < .05), while miR‐16 overexpression failed to significantly affect CAIF (Figure 4D). MSP was performed to analyze the effects of CAIF overexpression on miR‐16 gene methylation. Compared with cells transfected with the empty pcDNA3.1 vector, cells transfected with the CAIF expression vector showed significantly reduced miR‐16 gene methylation (Figure 4E).

### 3.5 LPS‐downregulated CAIF and miR‐16 expression in AC16 cells

**Figure 5:**
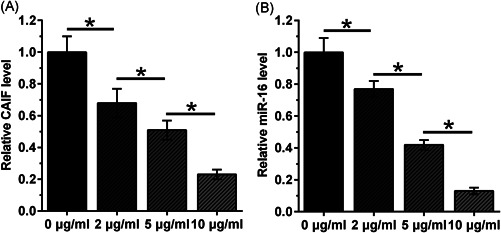
LPS‐induced CAIF and miR‐16 downregulation in AC16 cells. AC16 cells were incubated with LPS at doses of 0, 2, 5, and 10 µg/ml for 48 h. Thereafter, the expression levels of CAIF (A) and miR‐16 (B) were measured. The experiments were repeated in triplicate, and the mean ± *SD* values were compared. CAIF, cardiac autophagy inhibitory factor; LPS, lipopolysaccharide; miR, miRNA. *p < .05

AC16 cells were incubated with LPS at doses of 0, 2, 5, and 10 µg/ml for 48 h, followed by measurement of CAIF and miR‐16 expression levels. Both CAIF and miR‐16 were downregulated after LPS stimulation in a dose‐dependent manner (Figure 5A,B, *p* < .05). A 4.1‐fold decrease in CAIF (Figure 5A) and a 5.6‐fold decrease in miR‐16 (Figure 5B) were observed after treatment with 10 µg/ml LPS.

### 3.6 Both CAIF and miR‐16 inhibited LPS‐induced activation of the NF‐κB signaling pathway and inflammatory response

**Figure 6:**
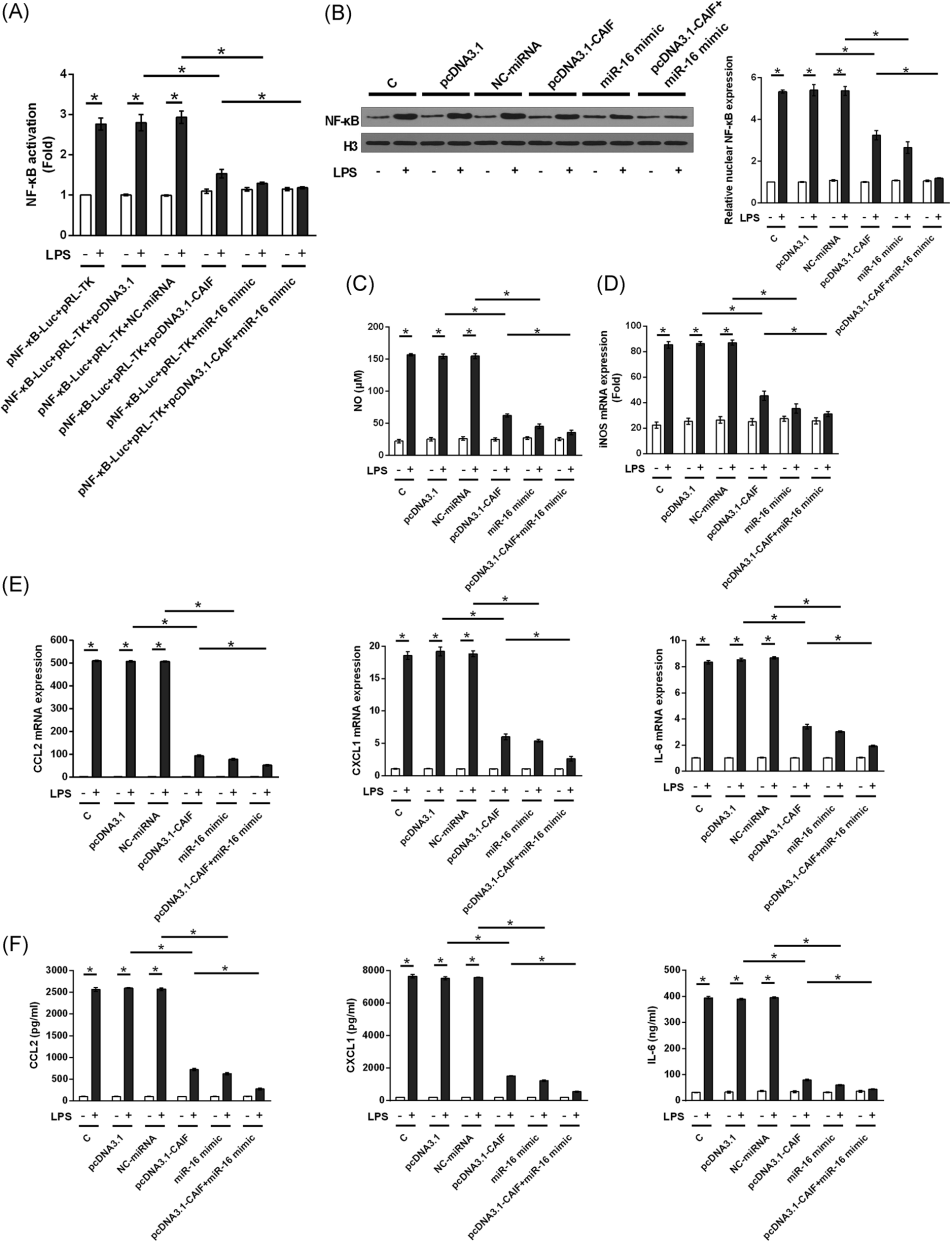
CAIF and miR‐16 inhibited LPS‐induced activation of the NF‐κB signaling pathway and inflammatory response. After transfection, AC16 cells were incubated with 5 µg/ml LPS for 24 h. Both CAIF and miR‐16 significantly inhibited LPS‐induced NF‐κB activation (A), NF‐κB expression (B), NO generation (C), and inducible nitric oxide synthase messenger RNA transcription (D). Both CAIF and miR‐16 inhibited LPS‐elevated CCL2, CXCL1, and IL‐6 expression at the miRNA (E) and protein (F) levels. C, control cells without transfection; CAIF, cardiac autophagy inhibitory factor; CCL2, C‐C motif chemokine 2; CXCL1, growth‐regulated alpha protein; IL, interleukin; LPS, lipopolysaccharide; miR, miRNA; NC, cells transfected with pcDNA3.1, or NC miRNA; NF‐κB, nuclear factor‐κB; NO, nitric oxide. *p < .05

AC16 cells were transfected with NF‐κB‐Luc + pRL‐TK, with or without pcDNA3.1, pcDNA3.1‐CAIF, NC‐miRNA, miR‐16, or pcDNA3.1‐CAIF + miR‐16 for 36 h before treatment with 5 µg/ml LPS. LPS‐induced activation of NF‐κB was significantly inhibited by CAIF and/or miR‐16 (Figure 6A, *p* < .05). Moreover, the LPS‐induced increase in NF‐κB expression was suppressed by CAIF and/or miR‐16 (Figure 6B, *p* < .05). Moreover, both CAIF and miR‐16 significantly inhibited the LPS‐induced inflammatory responses, including NO generation (Figure 6C, *p* < .05) and iNOS expression (Figure 6D, *p* < .05), as well as CCL2, CXCL1, and IL‐6 at both the mRNA (Figure 6E, *p* < .05) and protein levels (Figure 6F, *p* < .05). Furthermore, co‐overexpression of CAIF and miR‐16 showed a synergistic effect (Figure 5, *p* < .05).

### 3.7 CAIF and miR‐16 overexpression suppressed LPS‐induced cardiomyocyte apoptosis

**Figure 7:**
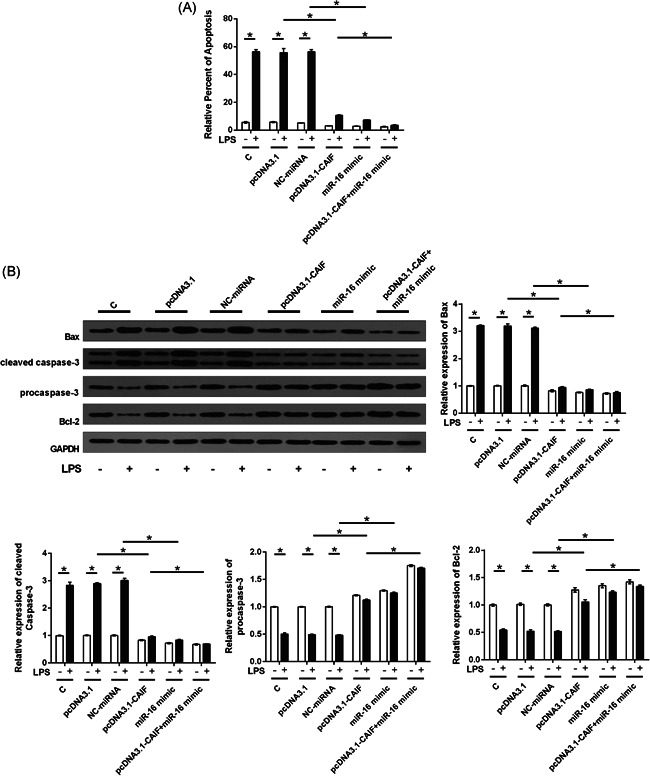
CAIF and miR‐16 overexpression suppressed LPS‐induced cardiomyocyte apoptosis. After transfection, AC16 cells were incubated with 5 µg/ml LPS for 24 h. A subsequent analysis of cell apoptosis (A), Bax, cleaved caspased‐3, procaspase‐3, and Bcl‐2 levels (B) was then performed. The experiments were repeated three times, and the mean ± *SD* values were compared. C, control cells without transfection; CAIF, cardiac autophagy inhibitory factor; LPS, lipopolysaccharide; miR, miRNA; NC, cells transfected with pcDNA3.1 or NC miRNA. *p < .05

After transfection, AC16 cells were incubated with 5 µg/ml LPS for 24 h. Thereafter, an analysis of cell apoptosis and related protein expression was conducted. Compared with non‐LPS treatment, LPS stimulation significantly increased AC16 cell apoptosis. However, CAIF overexpression and miR‐16 overexpression significantly inhibited the effect of LPS on AC16 cell apoptosis (Figure 7A, *p* < .05), suppressed Bax and cleaved caspase 3 levels, and promoted Bcl‐2 expression (Figure 7B, *p* < .05). In addition, co‐overexpression of CAIF and miR‐16 showed a synergistic effect (Figure 7, *p* < .05).

## 4 DISCUSSION

CAIF and miR‐16 have been reported to exhibit anti‐inflammatory effects in various inflammatory diseases. In the heart system, CAIF and miR‐16 have also been verified to play crucial and protective roles by targeting downstream proteins.^13^, ^14^, ^15^, ^16^, ^17^ In this study, the interaction between CAIF and miR‐16 was identified by bioinformatics and RNA pull‐down assays. In the subsequent experiments, we found that the expression levels of CAIF and miR‐16 were decreased in sepsis‐induced CHF. Further, a significant positive correlation was found between CAIF and miR‐16 in patient samples, but not in healthy controls, suggesting that this correlation only exists in a certain disease state, which may be in the inflammatory condition rather than in the normal condition.

CAIF has been demonstrated to inhibit inflammatory responses in osteoarthritis.^17^ However, it is not clear how CAIF changes in the inflammatory state. Our study found that CAIF expression in AC16 cells decreased with an increase in LPS concentration, consistent with our findings in human plasma that CAIF levels were lower in sepsis‐induced CHF patients than in healthy controls. MiR‐16 has also been found to have suppressive effects on inflammation in some diseases.^18^, ^19^, ^20^, ^21^ Our study found that, similar to CAI, miR‐16 expression was decreased in sepsis‐induced CHF patients. In addition, miR‐16 expression in AC16 cells decreased with increasing LPS concentration, suggesting that miR‐16 might be an inflammatory response resistance factor. Moreover, both CAIF and miR‐16 inhibited cardiomyocyte apoptosis. In addition, CAIF overexpression significantly increased miR‐16 expression in AC16 cells, while miR‐16 overexpression had no effect on CAIF expression. Furthermore, CAIF overexpression significantly reduced miR‐16 methylation. These results suggest that CAIF may suppress cardiomyocyte apoptosis and LPS‐induced inflammation by regulating miR‐16 demethylation.

The NF‐κB signaling pathway was most remarkably activated by LPS. Therefore, we examined whether CAIF and miR‐16 could influence this pathway. Both CAIF and miR‐16 overexpression significantly blocked LPS‐induced NF‐κB activation and expression, while miR‐16 downregulation reversed the effects of CAIF overexpression. The overexpression of CAIF and miR‐16 also inhibited NO generation and iNOS mRNA expression and suppressed CCL2, CXCL1, and IL‐6 expression, indicating the protective roles of CAIF and miR‐16 in counteracting inflammation.

A study reported that CAIF inactivated p53‐mediated transcription in the myocardia,^13^ suggesting that CAIF might inhibit cell apoptosis. Interestingly, our study showed that CAIF suppressed LPS‐induced cardiomyocyte apoptosis, reduced Bax and cleaved caspase 3 levels, and increased Bcl‐2 expression, indicating that CAIF may play a protective role in sepsis‐induced CHF by inhibiting cell apoptosis. These results provide potential novel molecular diagnostic biomarkers and treatment targets for patients with CHF. Although miR‐16 and CAIF play protective roles in sepsis‐induced CHF, their clinical value in CHF prognosis and treatment needs to be further evaluated. Notably, miR‐16 and CAIF have multiple downstream targets and can participate in different pathological and physiological processes. Thus, their clinical applications should be precisely evaluated. Importantly, we observed that CAIF and miR‐16 were positively correlated with each other only across plasma samples from sepsis‐induced CHF patients, but not from healthy controls. Further, we found that demethylation might be a pathway for miR‐16 regulation by CAIF. However, other factors that promote their interactions remain to be characterized. Based on the results of this study, we hope to contribute to clinical studies, and can preregain patients with CHF patients by detecting the expression of CAIF and miR‐16 in patients. CAIF could increase the expression of miR‐16 by increasing the expression of CAIF, thereby promoting inflammatory response, activating NF‐KB pathways, inhibits CCL2, INOS, improves the patient's immunity, thereby reaching a certain extent treatment CHF patient condition.

## 5 CONCLUSION

In conclusion, our study is the first to report the abnormal expression of CAIF and miR‐16 in heart disease. CAIF plays a protective role in sepsis‐induced CHF by inhibiting cardiomyocyte apoptosis and inflammation, possibly by regulating miR‐16 demethylation.

## CONFLICT OF INTERESTS

The authors declare that there are no conflict of interests.

## AUTHOR CONTRIBUTIONS

**Yan Wang**: wrote the manuscript and developed the study design. **Yi Zhang**: carried out literature search, data analysis, and statistical analysis. All authors read and approved the final manuscript.

## ETHICS STATEMENT

Ethical approval was obtained from the Ethics Committee of the Shaanxi Provincial People's Hospital (No. X1520113). All procedures involving human participants were performed in accordance with the 1964 Helsinki Declaration and its later amendments or comparable ethical standards.

## Data Availability

The datasets used and/or analyzed in the current study are available from the corresponding author upon reasonable request.
